# Temperature‐Driven Emission Redistribution of Yb^2+^ in SrB_4_O_7_ Enables Highly Sensitive Optical Thermometry Supported by Multiple Linear Regression

**DOI:** 10.1002/advs.75586

**Published:** 2026-05-08

**Authors:** Fang Zhao, Teng Zheng, Przemysław Woźny, Miguel A. Hernández‐Rodríguez, Shiqing Xu, Inocencio R. Martín, Marcin Runowski

**Affiliations:** ^1^ School of Information and Electrical Engineering Hangzhou City University Hangzhou Zhejiang China; ^2^ Faculty of Chemistry Adam Mickiewicz University Uniwersytetu Poznańskiego 8 Poznań Poland; ^3^ Departamento De Física IUdEA IMN and MALTA Consolider Team Universidad De La Laguna San Cristóbal de La Laguna Santa Cruz de Tenerife Spain; ^4^ Key Laboratory of Rare Earth Optoelectronic Materials and Devices of Zhejiang Province College of Optical and Electronic Technology China Jiliang University Hangzhou China

**Keywords:** decay time amplitudes, divalent lanthanide ions, excited states lifetime, luminescence thermometry, multiple linear regression (MLR)

## Abstract

Optical thermometry is a modern, non‐contact technique for temperature measurements, and its performance is fundamentally determined by the thermometric parameters employed. The use of unique luminescence from some divalent lanthanide ions may significantly boost the thermometric performance of optical sensors. Here, a first luminescent thermometer based on the rarely observed UV emission of Yb^2+^ is presented. The emitting lanthanide ions were successfully incorporated in the SrB_4_O_7_ host, whose thermal stability was confirmed in a broad *T*‐range of ≈80–420 K. The material subjected to extreme temperature conditions manifests luminescence enhancement and spectral shifts toward both higher and lower energies, depending on the *T*‐range studied. This is due to the thermalization processes within the excited electronic configuration 4*f* ^13^5*d* ^1^ of Yb^2+^, leading to distinct temperature responses of different relaxation channels. The applied multiple linear regression (MLR) analysis allows the combination of different thermometric parameters and a multiple increase of the resulting temperature sensitivity. Importantly, the proposed lifetime‐derived thermometric parameter, based on the decay‐amplitudes ratio, results in the unprecedentedly high relative temperature sensitivity of 27.64 % K^−1^. This approach provides a generalizable framework for extracting temperature information from luminescence dynamics in diverse phosphors, offering a new route for constructing high‐performance optical thermometers.

## Graphical abstract

A novel optical thermometer exploits the rare UV emission of Yb^2+^ ions in a SrB_4_O_7_ host, enabling highly sensitive, non‐contact temperature measurements from 80 to 420 K. The applied multiple linear regression (MLR) analysis results in a multiple increase in the resulting temperature sensitivity. The proposed thermometric parameter, based on the decay‐amplitudes ratio, results in the unprecedentedly high temperature sensitivity (27.64 %/K).

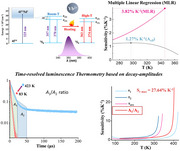

## 1 Introduction

Optical thermometry, as a non‐contact and non‐invasive temperature sensing technique, attracted great attention owing to its importance in micro‐ and nanoscale devices, extreme‐environment monitoring, and biomedicine systems [1, 2]. Compared with conventional contact‐based temperature sensors, optical thermometry offers great advantages, including fast response, high spatial resolution, and independence to electromagnetic interference [3, 4]. Recent remarkable advances in luminescence thermometry encompass the detection of local temperature fluctuations in catalytic reactors [5, 6], the use of luminescent thermometers for the vacuum level monitoring [7], the development of excitation‐light‐free optical temperature detection via mechanoluminescence [8, 9], and so forth. In addition to advances in luminescent materials, the performance of optical thermometers critically depends on the thermometric parameters employed for temperature readout [10, 11, 12].

The most common luminescence‐based thermometric strategies have been established on various thermometric parameters such as fluorescence intensity ratios, spectral shifts, bandwidth change, and luminescence lifetimes variation [9, 13, 14, 15]. Although these approaches have been widely adopted, each of them suffers from intrinsic limitations [16, 17]. For example, fluorescence intensity ratio‐based methods generally require well‐separated emission bands and are affected by errors from spectral overlap, reabsorption effects, and calibration conditions [18]. In contrast, spectral shift and bandwidth‐based thermometry demand high spectral resolution and a reliable fitting procedure [10]. Among these approaches, lifetime‐based thermometry is particularly attractive because it is inherently independent of excitation intensity and optical path variations. However, using a single decay constant or an averaged lifetime limits temperature sensitivity and operating range [17]. Moreover, a single luminescence parameter is often insufficient to fully capture the complex thermally induced relaxation processes in solid‐state emitters, as radiative and non‐radiative decay pathways may exhibit distinct temperature dependences [19]. Consequently, developing new lifetime‐based thermometric parameters that can amplify temperature sensitivity by exploiting luminescence dynamics themselves remains a key challenge in the field of optical thermometry.

Rare‐earth luminescent materials are ideal systems for the development of lifetime‐based thermometric parameters due to their well‐defined electronic structures and high photostability [20]. In solid‐state hosts, rare‐earth ions typically exist in trivalent or divalent states, among which Yb^2+^ has attracted increasing interest because of its parity‐allowed 4*f* ^13^5*d* ^1^→4*f* ^14^ transition [21]. In contrast to Yb^3+^ ions, where optical properties are dominated by the parity forbidden 4*f*‐4*f* transitions, the 5*d* excited states of Yb^2+^ are highly sensitive to the local crystal field symmetry and lattice vibrations [22]. As a result, both radiative and non‐radiative relaxation processes in Yb^2+^‐activated systems can be strongly modulated by temperature, thereby making these systems particularly suitable for the investigation of temperature‐dependent luminescence dynamics and for constructing new steady‐state and time‐resolved, i.e., lifetime‐based optical thermometers.

The application of Yb^2+^ dopant ions with unique optical features requires careful selection of a host lattice, being capable of stabilizing divalent lanthanide ions and effectively suppressing non‐radiative relaxation channels. Borate‐based compounds are widely investigated as excellent luminescent hosts for stabilization of rare‐earth ions at +2 oxidation state, mainly thanks to their high chemical and physical stability, unique 3D structure, rigid framework, and the presence of divalent cations (Sr^2+^) with suitable ionic radii [23, 24]. Among them, SrB_4_O_7_ possesses a very rigid 3D framework composed of corner‐sharing (BO_4_)^5−^ tetrahedra, which enables the stabilization of divalent lanthanide ions in the Sr^2+^ site under atmospheric conditions (i.e., in the presence of oxygen) [25]. Although SrB_4_O_7_ has been extensively studied as a host for some divalent lanthanide ions [26, 27], and their application in optical pressure and temperature sensing, as in the case of the Eu^2+^, Sm^2+^, or Tm^2+^ doped phosphors, there are neither reports on the luminescence of Yb^2+^ embedded in its crystal lattice nor the application of Yb^2+^ in optical thermometry.

In this work, we show the development of the first luminescent thermometer based on the SrB_4_O_7_:Yb^2+^ material, by exploiting the unique and rarely observed emissions from thermalized excited states of divalent Yb ions. Moreover, we used this material as a model system for multiple linear regression (MLR) analysis of intensity‐ and energy‐based spectroscopic parameters for improved thermometric performance, which enables the simultaneous utilization of multiple independent thermometric indicators and thus overcomes the limitations associated with conventional single‐parameter approaches. Its luminescence properties were investigated under variable temperature conditions (≈80–420 K), revealing significant temperature‐induced emission enhancement, spectral blue‐ and red‐shifts, and lifetimes shortening. In order to probe temperature‐induced changes in luminescence dynamics, we also analyzed the relative contribution of different emission decay components (decay‐amplitude ratios), introducing a new kinetic thermometric parameter that directly reflects the redistribution of excited‐state populations with temperature. By determining for the first time this thermometric parameter, the present approach provides a new framework to translate variations in excited‐state kinetics into an enhanced temperature readout, resulting in unprecedentedly high thermal sensitivity. These concepts, i.e., MLR and the use of decay amplitudes, establish new parameterization strategies for analyzing temperature‐dependent steady‐state and time‐resolved luminescence in phosphor materials, and enable the development of alternative routes toward high‐performance optical thermometry.

## 2 Results and Discussion

### 2.1 Properties at Ambient Condition

**FIGURE 1:**
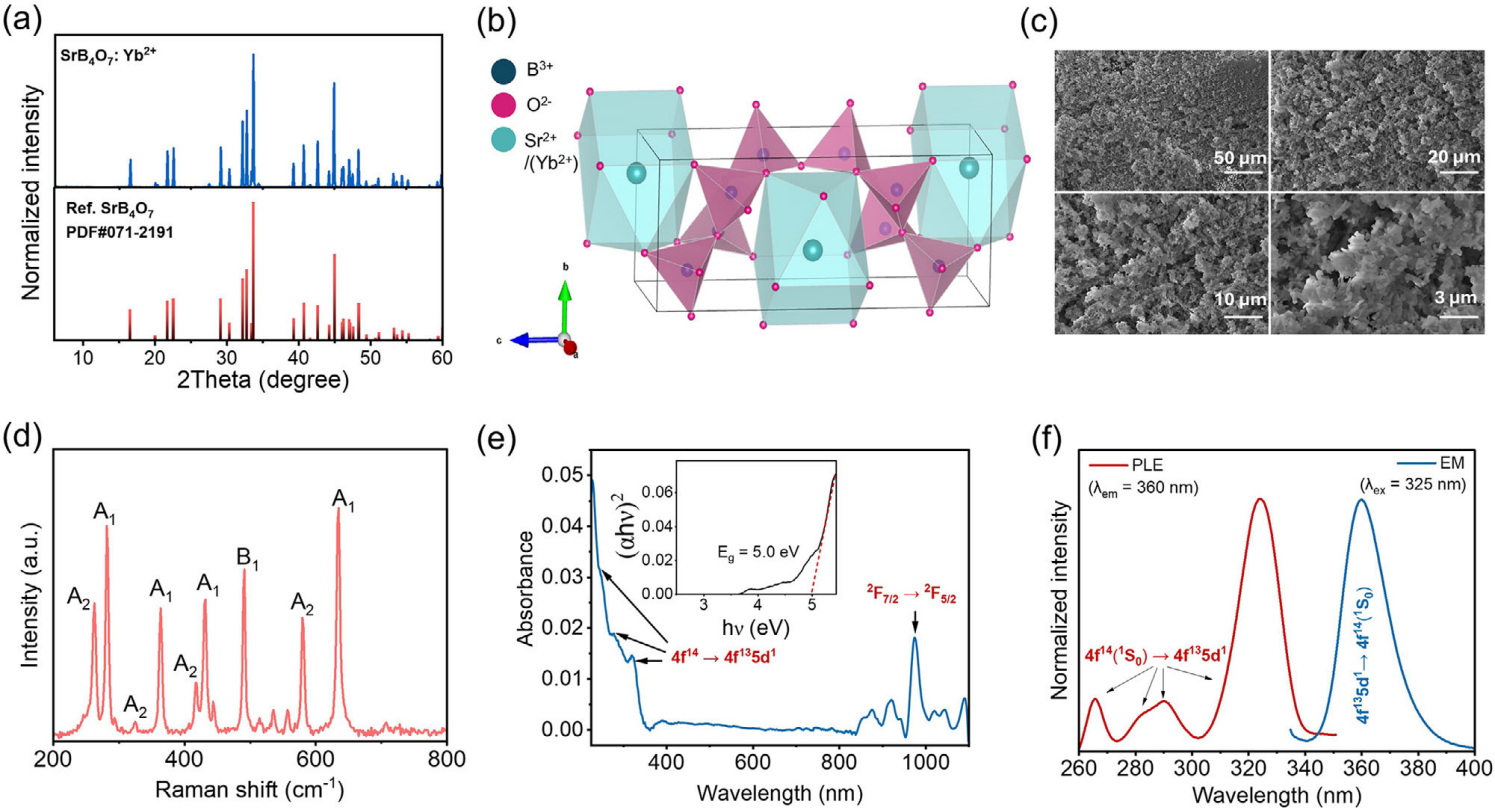
(a) XRD pattern of the SrB_4_O_7_:Yb^2+^ (top) compared with the corresponding standard reference card from the JCPDS database (bottom). (b) 3D representation of the SrB_4_O_7_:Yb^2+^ crystal structure. (c) SEM image of the synthesized sample at different magnifications. (d) Raman spectrum of the obtained material. (e) Absorption spectrum of the sample; the inset shows the corresponding Tauc plot, and the estimated band gap. (f) Excitation and emission spectra of the SrB_4_O_7_:Yb^2+^.

The XRD pattern of the synthesized SrB_4_O_7_:Yb^2+^ material is shown in Figure 1a (top). All diffraction reflexes can be indexed to the orthorhombic SrB_4_O_7_ phase (PDF#071‐2191, space group *Pmn2_1_*), with peak positions matching the reference pattern of the pure host (Figure 1a; bottom), indicating the expected crystal symmetry and lattice parameters of pure SrB_4_O_7_ material. The sharp and well‐defined peaks reflect well‐crystallized domains with good crystallinity and low microstrain. Upon Yb^2+^ doping, neither additional peaks nor noticeable shifts are detected, indicating that Yb^2+^ ions are incorporated into the SrB_4_O_7_ lattice, at Sr^2+^ sites, without forming impurity phases. Figure 1b presents the visualization of the SrB_4_O_7_ crystal structure, which consists of interconnected [BO_4_] tetrahedra forming a 3D chain framework [19]. Sr^2+^ ions in SrB_4_O_7_ are 9‐fold coordinated by O atoms, providing a suitable environment for isovalent substitution by Yb^2+^ ions. According to Shannon's ionic radii [28], although Yb^2+^ (1.30 Å) is slightly smaller than Sr^2+^ (1.31 Å), the difference in ionic radii is very low, indicating that the host lattice can effectively accommodate Yb^2+^ ions without inducing lattice distortion. The effective site symmetry for the Yb^2+^ dopant ions is *C_4v_* [7, 29]. Importantly, in this crystal structure, there is only one crystallographically independent site for Sr^2+^/Yb^2+^ ions [24, 26].

The SEM image (Figure 1c) reveals that the sample consists of irregular block‐like agglomerates. To obtain a more reliable particle size evaluation, the sizes of over 100 particles were measured, and the corresponding size distribution histogram with Gaussian fitting is presented in Figure S1. The particles are mainly distributed in the range of 0.1‐1.0 µm, with the majority centered around 0.5–0.6 µm (mean size ≈ 0.58 µm). Such submicrometer‐sized particles with relatively uniform distribution are beneficial for reducing excessive surface quenching, which is favorable for efficient photoluminescence. The corresponding EDX spectrum (Figure S2) confirms the presence of elements constituting the investigated material, i.e., Sr, B, O, and Yb. The optical properties were further investigated. The room‐temperature Raman spectrum of SrB_4_O_7_:Yb^2+^ is shown in Figure 1d. Several sharp vibrational bands are observed in the 200–700 cm^−1^ region, indicating the high crystallinity of the sample. The intense band located at ∼636 cm^−1^ is attributed to the symmetric stretching vibration (ν_1_) of BO_4_ tetrahedra, which is the characteristic fingerprint of orthoborate frameworks. Additional bands at ∼582, ∼492, ∼433, and ∼365 cm^−1^ are assigned to the bending vibrations of BO_4_ units and B‐O‐B linkages. More specifically, the major peaks originate from the A_1_ and A_2_ modes, and the minor ones come from the B_1_ and B_2_ modes, as indicated in the graph, which agrees well with the literature data [7, 30]. Notably, no obvious bands associated with BO_3_ units are detected in the higher wavenumber region, suggesting that the borate network is predominantly constructed from BO_4_ tetrahedra. Moreover, the absence of extra impurity peaks indicates that Yb^2+^ doping does not alter the host lattice structure.

The absorption spectrum (Figure 1e) exhibits two prominent bands centered at approximately 325 and 975 nm. The near‐UV band is attributed to the allowed 4*f* ^14^ → 4*f* ^13^5*d* ^1^ transition of Yb^2+^, while the weak near‐infrared absorption originates from the 4*f*→4*f* (^2^F_7/2_ → ^2^F_5/2_) transition of Yb^3+^. Notably, the Yb^3+^ absorption is located in the NIR region and does not spectrally overlap with the emission band discussed in this work and therefore does not affect the analysis of Yb^2+^ emission. Using the Tauc plot (Figure 1e, inset), the optical band gap of the SrB_4_O_7_ host was estimated to be approximately 5.0 eV, consistent with the wide‐bandgap nature of this borate material. Additionally, the performed x‐ray photoelectron spectroscopy (XPS) studies confirmed the presence and stability of Yb ions in the material studied in both valence states, i.e., as Yb^2+^ and Yb^3+^ (Figure S3), being in good agreement with the absorption data. It should be noted that mixed valence states of Yb ions are commonly observed in inorganic materials containing the reduced form of Yb [31, 32].

Next, the luminescence properties of the SrB_4_O_7_:Yb^2+^ were investigated, and the recorded photoluminescence excitation (PLE) and emission (PL) spectra are shown in Figure 1f. The excitation spectrum (red curve) consists of four bands (centered at around 266, 282, 290, and 324 nm), associated with transitions from the ground state of Yb^2+^ 4*f* ^14^ (^1^S_0_) to different states of its excited electronic configuration 4*f* ^13^5*d* ^1^ [33]. Specifically, in the case of the *C_4v_* site symmetry, this excited configuration should split due to crystal field effects into four components, i.e., *a*_1_(*z* ^2^) + *b*_1_(*x* ^2^‐*y* ^2^) + *b*_2_(*xy*) + *e*(*xz*, *yz*) [26], which agrees well with the recorded PLE spectrum. Under 325 nm excitation, a broad and intense emission band of Yb^2+^ is observed at approximately 360 nm, which corresponds to the radiative transition from the excited 4*f* ^13^5*d* ^1^ configuration to the ground‐state 4*f* ^14^ (^1^S_0_) of Yb^2+^, confirming the presence of Yb ions in divalent state in the SrB_4_O_7_ host lattice [33, 34]. The broad emission band reflects the strong sensitivity of the 5*d*‐4*f* transitions to the local crystal field environment. The above results imply that the SrB_4_O_7_ host matrix provides stable luminescence centers for Yb^2+^ ions.

### 2.2 Temperature Properties

**FIGURE 2:**
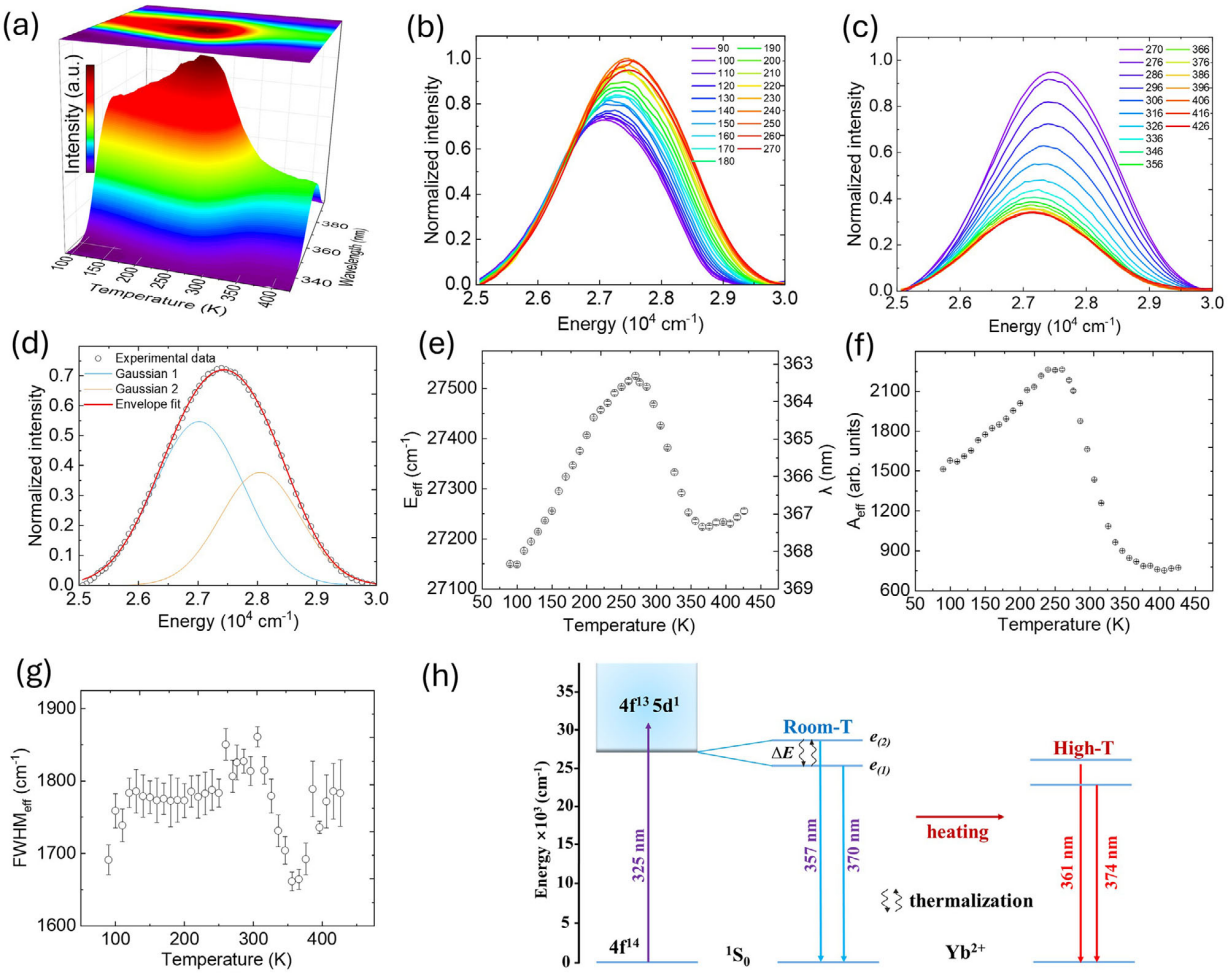
(a) 3D emission spectra of the SrB4O7:Yb^2+^ as a function of temperature. (b) Normalized Jacobian converted emission spectra within the low‐temperature range (90–270 K), and (c) the high‐temperature range (270–420 K). (d) Gaussian deconvolution of the emission spectrum for the SrB4O7:Yb^2+^ recorded at 296 K, implemented in a homemade Python code. Temperature‐dependent of the effective (e) peak energy (*E*_eff_), (f) area (*A*_eff_), and (g) FWHM (*W*eff). (h) Schematic energy‐level diagram showing the room‐temperature and high‐temperature radiative transitions within Yb^2+^ ions in the strontium borate host.

**FIGURE 3:**
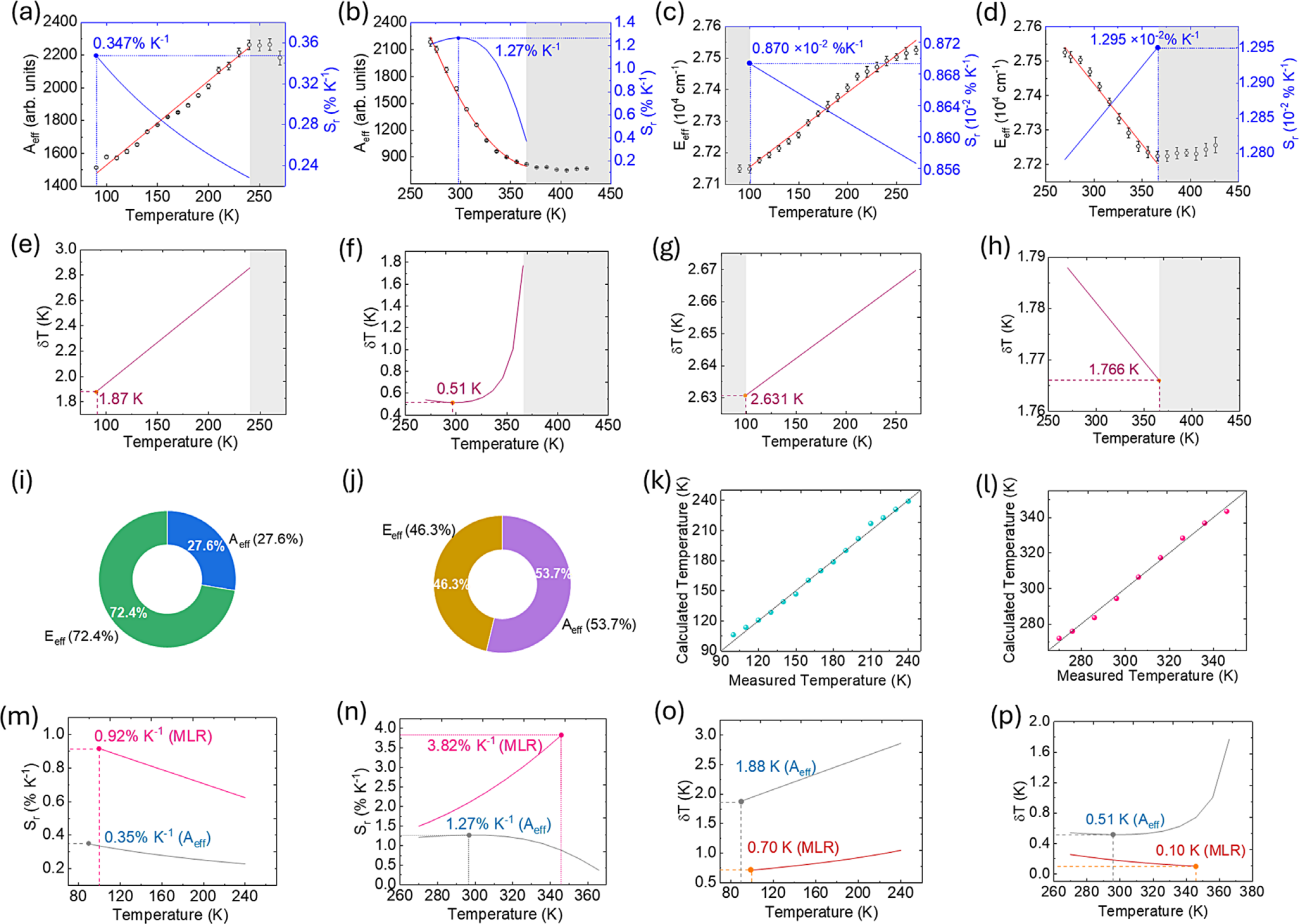
(a‐d) Temperature evolutions of the effective area—*A*_eff_ and peak energy—*E*_eff_ within the (a, c) 90–270 K and (b, d) 270–426 K temperature ranges respectively; the red solid lines in panels a, c and d, represent linear fits, and in the case of the b panel the quadratic function used as the best fit to the experimental data; the relative sensitivity—*S*_r_ is also indicated in all panels as blue solid lines with the corresponding maximal values; the grey shadowed regions in a‐d panels represent the temperature range where the optical thermometer becomes unreliable, as the uncertainty in the experimental data exceeds the signal difference between adjacent calibration points, making it impossible to resolve the temperature changes reliably. (e‐h) The associated temperature uncertainties—δ*T* for the effective areas—*A_eff_* (e, f) and effective peak energies—*E_eff_* (g, h), and the indicated minimum values. The grey shadowed regions in the e‐h panels represent the temperature range where the optical thermometer fails. (i, j) Doughnut charts of the relative weight (values in parenthesis are the *β*‐weights) of the thermometric parameters considered for the MLR analysis within the ranges of (i) 100–240 K and (j) 270–346 K. (k, l) Correlation between the measured temperature (*x*‐axis) and the calculated temperature from the combination of all the thermometric parameters (*y*‐axis) within the *T*‐ranges (k) 100–240 K and (l) 270–346 K. (m‐p) The S_r_ values (m, n) and the corresponding δ*T* (o, p), using the MLR approach. The gray solid lines in the panels (m, n) and (o, p) represent the S_r_ and δ*T* values computed using a single parameter approach, together with corresponding maximum and minimum values, respectively. The dashed lines are guides for the eyes corresponding to *y* = *x* (the MLR fit parameters are shown in Table S3).

To evaluate the potential of the SrB_4_O_7_:Yb^2+^ as an optical thermometer, its temperature‐dependent photoluminescence properties were investigated over a wide temperature range of ≈ 80–420 K. Figure 2a shows the emission spectra of the investigated material as a function of temperature, providing an overview of the continuous evolution of intensity and spectral position of the Yb^2+^ emission band at λ ≈ 360 nm. Due to the wide nature of this emission band, it is convenient to present the spectra and perform calculations in the energy domain instead of wavelength units. In this regard, a Jacobian transformation of the spectra was performed following procedures reported elsewhere [35, 36, 37]. For better clarity, the temperature evolution of the Jacobian‐transformed spectra within two temperature ranges, i.e., 90–270 K and 270–426 K, is shown in Figures 2b and 3c, respectively. The initial increase of the luminescence intensity with temperature, i.e., from 90 to 270 K (see Figure 2b), is due to the enhanced thermal population of the higher‐lying excited states with large radiative transition rates, which agrees well with the model implemented in this work, the performed kinetics studies, and literature reports [33]. The further intensity decrease is caused by the well‐established process of thermal quenching of luminescence under high‐temperature conditions.

At first glance of Figure 2b,c, the blue and red shifts can be observed in the first and second temperature ranges, respectively. However, for a more precise quantification of the temperature‐driven changes in the emission spectrum of the SrB_4_O_7_:Yb^2+^, a double Gaussian deconvolution fit was performed. In this analysis, two Gaussian components, labeled as 1 and 2, were used to represent different contributions of the emitting states of Yb^2+^ to the overall emission signal, as illustrated in Figure 2d. From the deconvolution fit, the parameters of each Gaussian component (*i* = 1,2) were determined, namely the peak energy (*E_i_*), area (*A_i_*), and the full‐width at half maximum – FWHM (*W_i_*), which are summarized in Table S1. Subsequently, these parameters were used to compute the corresponding effective parameters, i.e., the effective peak energy (*E_eff_* – spectral position of the band centroid), the effective area (*A_eff_* – integrated area under the curve), and band width (*W_eff_* = FWHM) following the procedure reported elsewhere [35, 36, 37]. These effective parameters are defined to provide a representative description of the overall luminescence response by accounting for the contribution of each Gaussian component. When emission bands are strongly overlapped, the area of each Gaussian, corresponding to its integrated intensity, provides a natural weighting factor that reflects the relative contribution of each component to the total emission profile. By employing area‐weighted averages, photophysical parameters such as peak position and bandwidth are represented proportionally to their actual spectral contributions. This approach ensures that the dominant components exert a greater influence on the effective values, resulting in a more accurate and physically meaningful characterization of the temperature‐dependent spectral behavior of the system under study.

The temperature dependences of the determined effective parameters are depicted in Figure 2e–g. On one hand, the temperature evolution of *E_eff_* confirms two different behaviors, i.e., a monotonic blue‐shift from 90 up to 270 K, followed by a linear red‐shift from this temperature up to 350 K, above which remains unchanged (see Figure 2e). On the other hand, a similar trend is inferred for the A_eff_, which increases approximately linearly up to ∼270 K, and then decreases at higher temperatures (Figure 2f). Although the *A_eff_* and *E_eff_* inflection points are not exactly identical, their proximity suggests that both parameters are likely governed by the same thermally activated redistribution of excited‐state population. In contrast, for *W_eff_*, a non‐monotonic and somewhat irregular temperature dependence is observed. Specifically, *W_eff_* increases from 90 to approximately 120 K, and remains nearly constant up to 270 K. It then decreases to about 350 K, followed by a partial recovery at higher temperatures. However, the significant scatter observed in this region indicates the absence of a clear systematic trend (see Figure 2g).

The dominant radiative transitions at low and high temperatures are schematically illustrated in the energy‐level diagram shown in Figure 2h. In the lower temperature range (90‐270 K), a moderate thermal activation populates the higher‐energy crystal field component of the excited 4*f* ^13^5*d* ^1^ electronic configuration of Yb^2+^, labeled as *e*_(2)_ state, which is thermally coupled to the lower‐energy component denoted as *e*_(1)_ state. Note, both states are separated by ≈10^3^ cm^−1^ (Δ*E*), as can be inferred from Figure 2d. The thermalization phenomenon enhances luminescence from higher‐energy states, resulting in a blue‐shifted emission accompanied by increased intensity. On the other hand, in the higher temperature range (90‐426 K), the enhanced electron‐phonon coupling and activation of non‐radiative decay channels dominate and deplete the higher excited states, leading to emission quenching and a shift of the emission centroid toward lower energies. Moreover, the energy separation between the excited and ground configurations decreases with temperature, which also contributes to the observed overall red shift of the emission band centroid. Our findings agree well with previous literature reports on Yb^2+^ emission under extreme conditions [33, 38].

To exclude structural phase transition or material decomposition under variable temperature conditions, the temperature‐dependent Raman spectra were recorded in the investigated *T*‐range (Figure S4). There are no traces of new Raman modes, and the spectra shape is preserved within the whole *T*‐range studied (Figure S4a). Moreover, we did not detect any anomalous rapid change in the temperature‐induced spectral shift of the peaks. The recorded bands exhibit monotonic shift toward lower wavenumbers, due to the thermal expansion of the lattice (bonds elongation), by around 2–3 cm^−1^, indicating that the SrB_4_O_7_ host remains structurally stable throughout the experimental temperature range. Whereas the observed thermal broadening is due to the enhanced anharmonic phonon‐phonon interactions, increased phonon population, and thermal expansion of the lattice.

### 2.3 Luminescence Thermometry in Steady State Conditions

#### 2.3.1 Single Parametric Analysis

The single‐parametric approach for optical sensing quantifies the performance of a given sensor by relying on a single measurable parameter to evaluate its response to external stimuli (e.g., temperature or pressure). For the assessment of the capabilities of SrB_4_O_7_:Yb^2+^ as an optical thermometer, the temperature dependence of *A_eff_* and *E_eff_* was analyzed within two distinct temperature ranges, as discussed above, i.e., below and above 270 K, respectively. The parameter *W_eff_* was excluded from this analysis due to its irregular temperature dependence and lack of a clear systematic trend. For both effective parameters, the calibration procedure was based on phenomenological fitting. On one hand, a linear function and a second‐order polynomial were employed as calibration curves in the 90–270 K and 270–426 K temperature ranges for *A_eff_*, respectively, as shown in Figure 3a,b. On the other hand, the temperature dependence of *E_eff_* was fitted using linear functions in both temperature intervals, as depicted in Figure 3c,d.

Based on these calibration curves, the main figures of merit used to evaluate the performance of the optical thermometer were determined, namely the relative temperature sensitivity (S_r_) and the temperature uncertainty (δT). The S_r_ value describes the ability of the thermometric parameter to respond to temperature variations (in % [T] units) and is defined as:

(1) $$S_{r}=\frac{1}{\Delta \left(T\right)}\left|\frac{\partial \Delta \left(T\right)}{\partial T}\right|$$

where Δ(*T*) represents the thermometric parameter (*A_eff_* or *E_eff_*). Whereas the δ*T*, which reflects the smallest detectable temperature change, is given by:

(2) $$\delta T=\frac{1}{S_{r}}\frac{\delta \Delta \left(T\right)}{\Delta \left(T\right)}$$

where δΔ(*T*)/Δ(*T*) is the experimental uncertainty in the determination of Δ(T). In the present study, the relative error was estimated by propagating the uncertainties with the parameters involved in the definition of Δ(*T*). In this context, the temperature dependence of the S_r_ value is also shown in Figure 3. The maximum S_r_ values achieved for the *A_eff_* are 0.34 and 1.27% K^−1^ in the temperature ranges below (see Figure 3a) and above 270 K (Figure 3b), respectively. In the case of the *E_eff_*, the maximum S_r_ values are 0.820 × 10^−2^ and 1.295 × 10^−2^% K^−1^ within the temperature intervals below (see Figure 3c) and above 270 K (Figure 3d). The corresponding minimum δT values are 1.87 and 0.51 K for the *A_eff_* (Figure 3e,f), and 2.63 and 1.77 K for the *E_eff_* (Figure 3g,h). The fitting parameters are given in Table S2.

Due to the non‐monotonic temperature dependence of both *A_eff_* and *E_eff_* over the full investigated range, these parameters cannot provide an unambiguous temperature readout in practical applications. For this reason, and in order to address this limitation, the calibration was performed separately in two temperature intervals, where the dependence remains monotonic and ensures a single‐valued and reliable temperature determination.

#### 2.3.2 Improved Thermometric Performance Using Multiple Linear Regression (MLR)

Recent studies have demonstrated comprehensive applications of multiple linear regression (MLR) in the field of optical sensing [37, 39, 40, 41]. This numerical approach aims to maximize relative sensitivity while minimizing the associated uncertainty by combining multiple features that depend on the measurable external stimulus, such as temperature or pressure. This approach was first introduced in the context of luminescence thermometry through the application of MLR [39], which enables the evaluation of the contribution of multiple independent variables to a single experimental response by integrating information from several thermometric parameters. A landmark example was reported in 2021 by Maturi et al. [40], who achieved a tenfold improvement in the performance of multiparametric nanothermometers based on Ag_2_S nanocrystals compared with conventional single‐parameter approaches. Notably, their study established one of the highest reported relative sensitivities (S_r_ ≈ 50% K^−^^1^) during in vivo measurements.

More recently, Martínez‐Merino et al. [41]. applied multiparametric MLR analysis to WSe_2_ quantum dot–based luminescent thermometers, reporting a remarkable increase in S_r_ from 0.8% to 30% K^−^^1^, further demonstrating the potential of this methodology for advanced optical sensing applications. Another example of the utility of MLR in the context of luminescent thermometry was reported by Samuel Vega et al. [42], in which the temperature performance of a dual‐mode optical sensor based on core‐shell silica particles functionalized with Rhodamine B and fluorescein isothiocyanate was assessed, improving roughly twofold the S_r_ compared to the single parametric sensing approach.

In parallel, this technique has recently been extended to luminescent manometry, specifically in cerium‐doped yttrium aluminum garnet (YAG:Ce^3^^+^) crystals. By combining different spectral features of the characteristic broadband emission, such as band energy and integrated intensity, a 50‐fold increase in relative sensitivity (50.8% GPa^−1^) and a fourfold reduction in pressure uncertainty (∼0.04 GPa) were achieved, marking a significant advance in multiparametric optical pressure sensing [37]. The MLR algorithm can be defined as:

(3) $$y=\beta_{0}+\sum_{i=1}^{n}\beta_{i}x_{i}+\varepsilon$$

where *y* represents the dependent variable (in this study, temperature), *x*_i_ are the independent variables, also referred to as predictors, *β*_i_ are the regression coefficients associated with each predictor, and ε denotes the residual random error. The regression coefficients quantify the variation in the dependent variable resulting from a one‐unit change in the corresponding predictor, while the remaining predictors are kept constant. The error term accounts for the unobserved factors influencing the dependent variable that are not explicitly included in the model.

In the present work, the predictors correspond to the effective spectroscopic parameters *A_eff_* and *E_eff_*, while temperature (*T*) is treated as the response variable. To ensure the validity of the linear regression model, the selected predictors were required to satisfy two main criteria: (i) they must exhibit a linear dependence on temperature, and (ii) they must share the same operational temperature range. Accordingly, two temperature intervals, i.e., below and above 270 K, were analyzed separately. Figure 3i–p presents the MLR analysis of the SrB_4_O_7_:Yb^2+^ below and above 270 K. Two selected thermometric parameters, namely *A_eff_* and *E_eff_*, exhibit linear dependence with temperature and share the same operational ranges, namely from 100 to 240 K and from 270 to 346 K. The combination of these two predictors was carried out following the procedure described in the literature, and the corresponding *β*‐coefficients were calculated [37, 40]. In the temperature range of 100–240 K, *E*_eff_ presents a higher *β*‐weight than *A*_eff_ (72.4% and 27.6%, respectively). According to the model, *E_eff_* plays a more significant role in the estimation of the temperature within this interval for a given value of *A_eff_* (see Figure 3i). In contrast, within the 270 – 346 K range, both *E_eff_* and *A_eff_* exhibit comparable *β*‐weights, meaning that both parameters contribute similarly to the temperature prediction in this range (see Figure 3j). The good correlations between the measured temperatures and those predicted by the MLR model are depicted in Figures 3k, l.

As previously reported, this method, in principle, can increase the thermometric performance of the SrB_4_O_7_:Yb^2+^ sensor material. The temperature dependence of the *S*_r_ and δ*T* is shown in Figure 3m–p respectively. The MLR model yielded maximum *S*_r_ values of 0.92% and 3.82% K^−^^1^ in the 100–240 K and 270–346 K temperature intervals, respectively, representing approximately a threefold improvement compared with the *S*_r_ values obtained from single‐parametric sensing in the same ranges (for *A*_eff_ it was 0.35% K^−^^1^ and 1.27% K^−^^1^). Additionally, the minimum δ*T* values achieved were 0.70 and 0.10 K, compared with 1.87 and 0.51 K obtained using the single‐parameter approach, respectively.

Although the improvement of the thermometric performance of the SrB_4_O_7_:Yb^2+^ sensor through MLR is not as spectacular compared to other MLR‐based optical sensors reported in the literature, the improvement is nevertheless evident, underscoring the straightforward implementation of this numerical method in any luminescent material for sensing applications, including temperature [20, 40, 42], pressure [37], or other measurable physical parameters.

### 2.4 Luminescence Thermometry in Non‐Steady State Conditions

**FIGURE 4:**
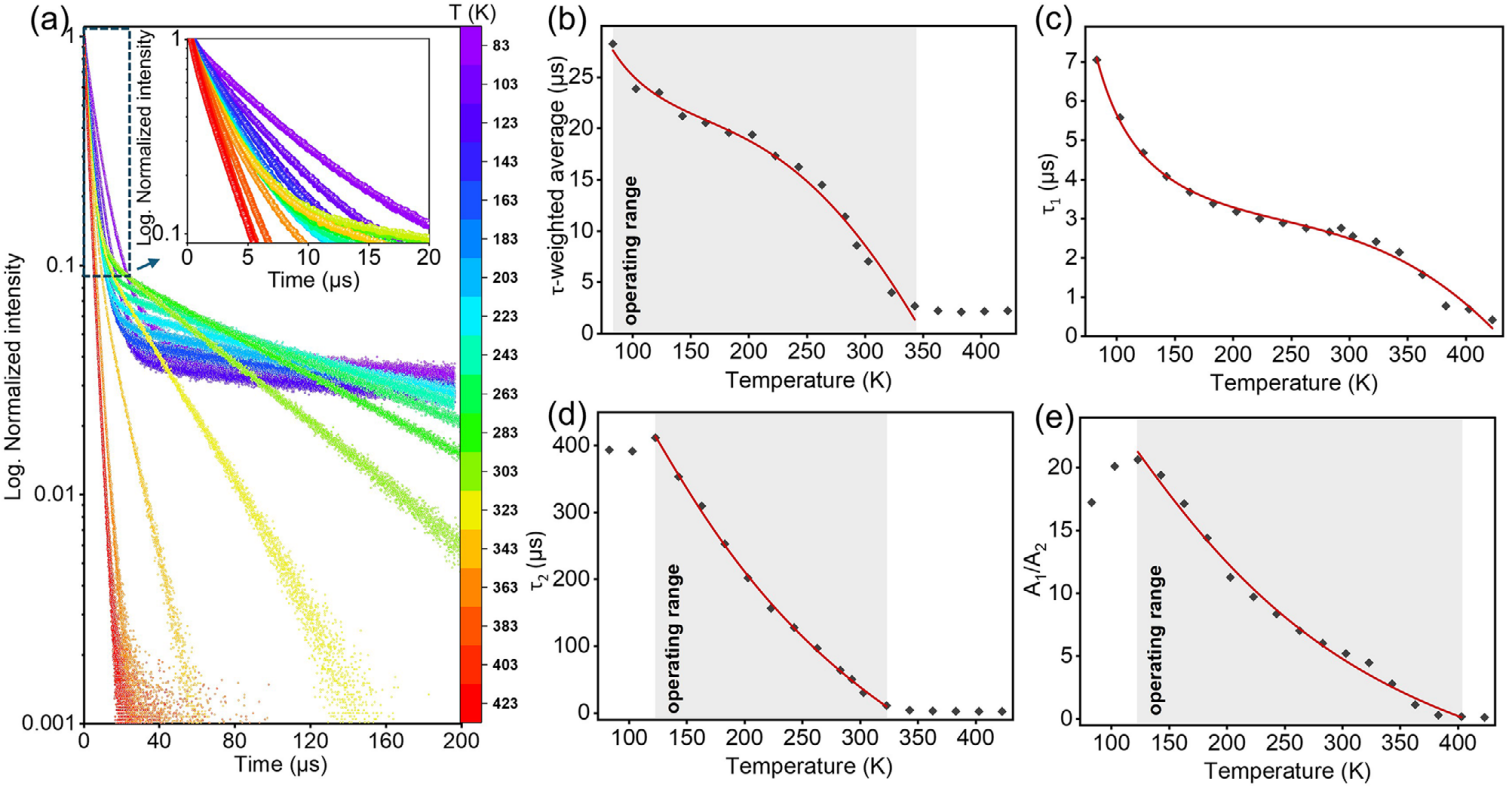
(a) Time‐resolved emission decay curves of the SrB_4_O_7_:Yb^2+^ over ≈83‐423 K (λ_ex_ = 325 nm; λ_em_ = 360 nm); the inset shows a magnified view of the 0–20 µs time frame of the decay curves. (b‐e) Temperature evolution of the derived kinetic parameters, i.e., *τ*_ave_ (b), *τ*_1_ (c), *τ*_2_ (d), and the ratio *A*_1_/*A*_2_ (e); the red lines represent the fitting curves.

To further explore the temperature response from a dynamical perspective, time‐resolved luminescence measurements were performed over a *T*‐range of 83 – 423 K (λ_ex_ = 325 nm; λ_em_ = 360 nm). Figure 4a presents the emission decay curves recorded at different temperature values, indicating their clear bi‐exponential character, and much faster decay rate at elevated temperature. All decay curves were well fitted using a bi‐exponential function (*R* ^2^>0.99), i.e.:

(4) I=A1exp(−x/τ1)+A2exp(−x/τ2)

where *I* is the luminescence intensity at time *x*, *A*_1_ and *A*_2_ are the corresponding pre‐exponential factors (amplitudes), *τ*_1_ and *τ*_2_ are short and long lifetime components. The average lifetime (*τ*_ave_) was calculated using the amplitude‐weighted method according to the formula:

(5) $$\tau_{ave}=\frac{A_{1}\tau_{1}^{2}+A_{2}\tau_{2}^{2}}{A_{1}\tau_{1}+A_{2}\tau_{2}}$$

As shown in Figure 4b–d, the *τ*_1_, *τ*_2_, and *τ*_ave_ decrease monotonically with increasing temperature, due to the temperature‐governed non‐radiative relaxation processes, and the already mentioned thermal population of high‐energy excited states with high radiative transition rates [33]. Their behavior can be described with the thermal activation model within the operating *T*‐range, using the following fitting function:

(6) $$\rho =\frac{1}{\tau }=\frac{1}{\tau_{0}}+\sum_{i=1}^{2}B_{i}\times exp\left(-\frac{\Delta E_{ai}}{k_{B}T}\right)$$

where *τ*_0_ is the intrinsic radiative lifetime at low temperature, *B_i_* is the rate coefficient of the *i*‐th non‐radiative channel, Δ*E_ai_* is the corresponding thermal activation energy, *k_B_* is the Boltzmann constant, and *T* is the absolute temperature. The fitting parameters and detailed values are listed in Table S4. The bi‐exponential model provides a good fit to all decay curves, indicating that the observed luminescence decay is well captured by two distinct relaxation channels. This observation agrees well with the model proposed in this work, i.e., the emission from two different excited states of Yb^2+^ in the SrB_4_O_7_ host, where the lanthanide dopant ions occupy only a single site.

Additionally, for the potential application in optical thermometry, we analyzed the temperature dependence of the corresponding amplitudes (*A_1_* and *A_2_*), which quantify the relative contributions of different relaxation channels to the overall emission. Based on this assumption, the ratio of the pre‐exponential factors from the bi‐exponential fit (*A*_1_/*A*_2_) was introduced as a new kinetic‐based thermometric parameter. As shown in Figure 4e, *A*_1_/*A*_2_ exhibits a pronounced and continuous response to temperature and can be accurately described by the following empirical function:

(7) $$A_{1}/A_{2}=B_{2}+\frac{B_{1}-B_{2}}{1+{\left(T/T_{0}\right)}^{c}}$$

where *B*_1_ and *B*_2_ represent the fitting coefficients, *T*_0_ is the initial temperature value, and *c* is an exponential factor. The fitting parameters are provided in Table S4. The fitted curve reproduces the experimental data well, showing a continuous decrease of the ratio *A*_1_/*A*_2_ with increasing temperature. This trend reflects the gradual change in the relative contributions of the short (*τ*_1_; *A*_1_) and long lifetime components (*τ*_2_; *A*_2_), indicating that the long component contributes in a larger extent at higher temperatures, and thus it affects more the overall decay dynamics.

**FIGURE 5:**
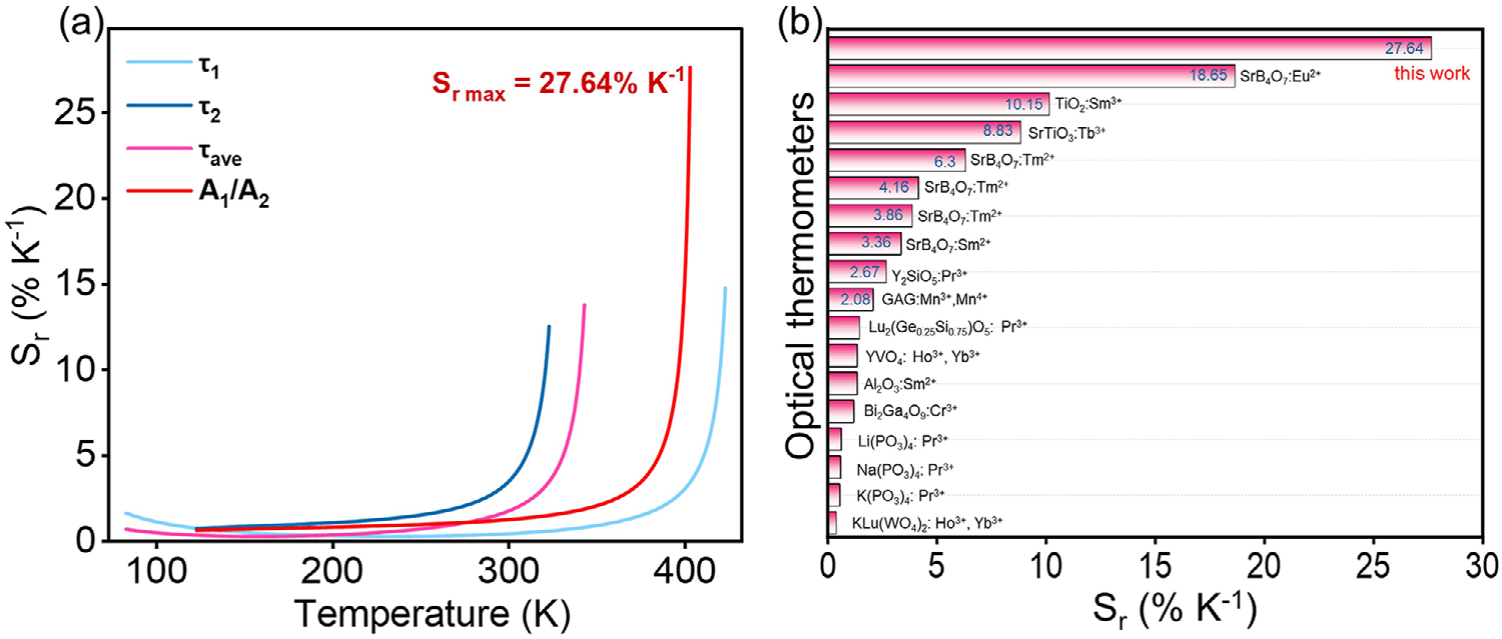
(a) Relative temperature sensitivity (S_r_) for the kinetic thermometric parameters *τ*_1_, *τ*_2_, *τ*_ave_, and *A*_1_/*A*_2_. (b) Comparison of the maximal S_r_ values for the reported optical thermometers based on photoluminescence kinetics.

Using the determined, four independent kinetic parameters, i.e., *τ*_1_, *τ*_2_, *τ*_ave_, and *A*_1_/*A*_2_, the thermometric performance of the SrB_4_O_7_:Yb^2+^ material was quantified in terms of the relative temperature sensitivity—*S_r_*. The *S_r_* values were calculated using Equation 1, by substituting the thermometric factor Δ(T) with a given kinetic parameter (*τ*_1_, *τ*_2_, *τ*_ave_, or *A*_1_/*A*_2_). For *τ*_1_, *τ*_2_, and *τ*_ave_ the *S_r_* values are initially (i.e. within the cryogenic range) about 0.5%–1%/K, and they start to increase at around room temperature, reaching the maximal values of 14.74%/K (at 423 K), 12.51%/K (at 323 K) and 13.77%/K (at 343 K), respectively. Unlike conventional lifetime thermometry, which relies on the temperature dependence of emission lifetime components (*τ*), the coefficient ratio *A*_1_/*A*_2_ directly probes the temperature‐driven redistribution of excited‐state populations between competing relaxation pathways. As a result, even a small thermal perturbation induces a pronounced change in the *A*_1_/*A*_2_ ratio, giving rise to an exceptionally high *S_r_* of 27.64% K^−1^ at 403 K (Figure 5a), significantly exceeding those of traditional lifetime‐based thermometric parameters. Figure 5b compares the performance of the investigated SrB_4_O_7_:Yb^2+^ material with representative lanthanide‐doped lifetime‐based optical thermometers reported in the literature [43, 44, 45, 46, 47, 48, 49, 50, 51, 52, 53, 54, 55]. Within this framework, *A*_1_/*A*_2_‐based thermometric parameter of the SrB_4_O_7_:Yb^2+^ shows superior sensitivity compared to the traditional lifetime‐based contactless thermometers. This result highlights a new operating principle for optical thermometry: exploiting thermally induced population redistribution among multiple emissive channels can surpass the intrinsic sensitivity limits of conventional lifetime‐based approaches.

Although decay‐amplitude ratios may provide high apparent sensitivities, it should be noted that the pre‐exponential coefficients obtained from multi‐exponential fitting can depend on the fitting model, the selected temporal window, and the signal‐to‐noise conditions of the measurement. In multi‐component decay analysis, the amplitudes are often correlated with the corresponding lifetimes and may vary without substantially affecting the overall quality of the fit. As a result, parameters derived from amplitude ratios may reflect not only intrinsic photophysical processes but also, to some extent, numerical aspects of the fitting procedure. In this context, the present results can be regarded as a proof‐of‐concept demonstrating the potential of exploiting decay‐dynamics features for thermometric applications. At the same time, this thermometric parameter is obtained from a model‐based fitting procedure and therefore benefits from consistent analysis conditions. Overall, the results indicate that decay‐amplitude ratios can provide a promising route for improvement of thermometric performance, while further assessment of their robustness and reproducibility would be of interest.

## 3 Conclusions

In summary, the luminescent thermometer based on Yb^2+^ emission has been developed for the first time, in the form of the SrB_4_O_7_:Yb^2+^ phosphor emitting in the UV range (around 360 nm). It was demonstrated as an effective model system for advancing traditional steady state luminescence thermometry based on intensity and band shift, by applying multiple linear regression (MLR) analysis, as well as for time‐resolved optical thermometry through the rational engineering of decay‐dynamic parameters. Owing to the strong coupling between the Yb^2+^ excited states of its electronic configuration 4*f* ^13^5*d* ^1^ and lattice vibrations, crystal field effects, and thermalization processes, the increasing temperature induces pronounced changes in its luminescence features, including intensity enhancement, spectral shifts, and significant changes in the emission kinetics. Specifically, in this case, temperature induces redistribution of radiative and nonradiative relaxation pathways, which manifests not only as lifetime shortening but also as a marked evolution of the relative decay amplitudes. By introducing for the first time the ratio of decay amplitudes (*A*_1_/*A*_2_) as a new thermometric parameter, temperature information is extracted directly from the thermally driven redistribution of excited‐state populations, thereby amplifying subtle thermal effects embedded in luminescence dynamics. As a result, the *A*_1_/*A*_2_ ‐based thermometry achieves a broad operating range of 120–400 K and an exceptionally high maximal sensitivity of 27.64% K^−1^, significantly surpassing conventional lifetime‐based approaches. This work demonstrates that the combination of advanced MLR analysis with rational exploitation of luminescence decay dynamics is a very powerful strategy for high‐performance, multiparametric optical thermometry and establishes a generalizable paradigm for divalent rare‐earth systems with thermally sensitive 5d states.

## 4 Experimental Section

### 4.1 Material Synthesis

All chemical reagents used in this study were of analytical grade with purities above 99.9% and were used without further purification. The phosphor material, i.e. SrB_4_O_7_ doped with 1 mol% of Yb^2+^, was synthesized by a conventional solid‐state reaction in an air atmosphere. In order to obtain 1 g of the SrB_4_O_7_:Yb^2+^ material, stoichiometric amounts of SrCO_3_, H_3_BO_3_, and Yb_2_O_3_ were thoroughly mixed and ground in an agate mortar, followed by a preliminary calcination at 750°C for 5 h to form the precursor. The pre‐reacted powders were then reground and subsequently sintered twice at 850°C for 5 h each to obtain the final product.

### 4.2 Characterization

Phase identification was performed using x‐ray diffraction (XRD) analysis with a Bruker AXS D8 Advance diffractometer, operating at Cu Kα_1_ radiation (λ = 0.1506 nm). The morphology and elemental composition were examined by scanning electron microscopy (SEM) and energy‐dispersive x‐ray (EDX) analysis, using FEI Quanta 250 FEG microscope equipped with an EDAX detector. Excitation and emission spectra at ambient conditions were recorded using a Hitachi F‐7000 fluorescence spectrophotometer equipped with a 150 W Xe lamp. Emission spectra under modified temperature were collected with a spectrometer (Andor Shamrock 500) equipped with a silicon CCD camera, and a with 325 nm UV diode (20 mW) used as an excitation source. Luminescence decay curves were acquired with a monochromator (TRIAX 180) with a photomultiplier connected to an oscilloscope, and a tunable ns‐pulsed laser EKSPLA/NT342/3/UVE (optical parametric oscillator – OPO), working at 10 Hz repetition rate (pulse duration of ≈7–8 ns) as an excitation source. Temperature‐dependent photoluminescence spectra and emission decay curves were recorded using the temperature‐controlled heating‐cooling stage (Linkam THMS600) to ensure precise and stable temperature regulation.

### 4.3 Multiple Linear Regression

The multiple linear regression (MLR) procedure was implemented using a built‐in function in MATLAB 2025a (licensed to the University of La Laguna). As predictors of temperature, *A*_eff_ and *E*_eff_ (*n* = 2) were taken from the emission spectra.

## Conflicts of Interest

The authors declare no conflicts of interest.

## Supporting information




**Supporting File**: advs75586‐sup‐0001‐SuppMat.docx

## Data Availability

The data that support the findings of this study are available from the corresponding author upon reasonable request.
