# Supplementary material for: Temperature‐Driven Emission Redistribution of Yb2+ in SrB4O7 Enables Highly Sensitive Optical Thermometry Supported by Multiple Linear Regression
Source: Adv Sci (Weinh). 2026 May 8;13(43):e75586. doi: 10.1002/advs.75586 (PMC13336091; doi:10.1002/advs.75586)
Supplement: Supplementary file 1 — Supporting File: advs75586‐sup‐0001‐SuppMat.docx [file ADVS-13-e75586-s001.docx]

**Supporting information for:**

**Temperature-Driven Emission Redistribution of Yb^2+^ in SrB_4_O_7_ Enables Highly Sensitive Optical Thermometry Supported by Multiple Linear Regression**

Fang Zhao, Teng Zheng^*^, Przemysław Woźny, Miguel A. Hernández-Rodríguez, Shiqing Xu^*^, Inocencio R. Martín, Marcin Runowski^*^

F. Zhao, T. Zheng, S. Xu

School of Information and Electrical Engineering, Hangzhou City University, Hangzhou, Zhejiang, China

F. Zhao, P. Woźny, M. Runowski

Adam Mickiewicz University, Faculty of Chemistry, Uniwersytetu Poznańskiego 8, 61-614 Poznań, Poland

M. A. Hernández-Rodríguez, I. R. Martín

Departamento de Física, IUdEA, IMN and MALTA Consolider Team, Universidad de La Laguna, San Cristóbal de La Laguna, Santa Cruz de Tenerife, E-38200, Spain

S. Xu

Key Laboratory of Rare Earth Optoelectronic Materials and Devices of Zhejiang Province, College of Optical and Electronic Technology, China Jiliang University, Hangzhou, 310018 China

**Corresponding Authors**

T.Z.: E-mail: [zhengteng@hzcu.edu.cn](mailto:zhengteng@hzcu.edu.cn)

X.S.: E-mail: [shiqingxu@cjlu.edu.cn](mailto:shiqingxu@cjlu.edu.cn)

M.R.: E-mail: [runowski@amu.edu.pl](mailto:runowski@amu.edu.pl)

**Table S1** Parameters retrieved from the Gaussian deconvolution fit of the broad emission band at different temperatures for SrB_4_O_7_:Yb^2+^.

|  | Parameters | | | | | |
| --- | --- | --- | --- | --- | --- | --- |
| T (K) | **A_1_**  **(±7.5 a.u.)** | **A_2_**  **(±14 a.u.)** | **E_1_**  **(±70 cm^-1^)** | **E_2_**  **(±60 cm^-1^)** | **W_1_**  **(±29 cm^-1^)** | **W_2_**  **(±30 cm^-1^)** |
| 90 | 3633.74047 | 465.63529 | 27043.96 | 27974.67 | 1788.18474 | 935.05865 |
| 100 | 3842.27053 | 447.04474 | 27051.35 | 27986.06 | 1854.05258 | 941.40657 |
| 110 | 3775.06615 | 514.40822 | 27066.61 | 27978.96 | 1837.27691 | 1016.50501 |
| 120 | 3949.42021 | 470.52447 | 27096.74 | 28013.27 | 1875.42807 | 1012.52684 |
| 130 | 3946.11166 | 584.76333 | 27097.18 | 28002.38 | 1884 | 1123.38194 |
| 140 | 4082.06549 | 696.56768 | 27103.14 | 28016.53 | 1884 | 1164.57614 |
| 150 | 4161.41971 | 702.98211 | 27117.9 | 28070.86 | 1884 | 1146.50185 |
| 160 | 4051.08795 | 963.75232 | 27118.11 | 28037.61 | 1884 | 1307.86541 |
| 170 | 4055.54601 | 1090.69928 | 27123.3 | 28068.38 | 1884 | 1372.23268 |
| 180 | 3953.9584 | 1327.78768 | 27108.12 | 28055.86 | 1884 | 1441.13242 |
| 190 | 4336.55865 | 1018.47968 | 27188.98 | 28168.49 | 1884 | 1304.54506 |
| 200 | 4353.78224 | 1288.5945 | 27181.24 | 28167.53 | 1884 | 1398.42108 |
| 210 | 4046.3342 | 1999.00278 | 27124.67 | 28083.43 | 1884 | 1586.00911 |
| 220 | 4625.93977 | 1406.23013 | 27215.81 | 28249.83 | 1884 | 1430.16698 |
| 230 | 4386.9944 | 1793.5507 | 27180.55 | 28181.44 | 1884 | 1534.74315 |
| 240 | 4157.91174 | 2313.29484 | 27138.15 | 28124.46 | 1884 | 1614.74589 |
| 250 | 4023.79174 | 2504.4261 | 27115.96 | 28124.49 | 1884 | 1622.15373 |
| 260 | 2986.3151 | 3708.06402 | 26963.7 | 27958.31 | 1884 | 1823.51157 |
| 270 | 3912.93752 | 2326.03015 | 27154.53 | 28148.23 | 1884 | 1676.44466 |
| 276 | 3341.55583 | 2738.48451 | 27069.77 | 28051.38 | 1884 | 1754.74047 |
| 286 | 3133.984 | 2369.26052 | 27076.26 | 28068.04 | 1884 | 1752.57388 |
| 296 | 2776.47351 | 2012.70357 | 27053.4 | 28041.53 | 1884 | 1717.03066 |
| 306 | 2075.02187 | 2127.63431 | 26933.94 | 27905.39 | 1873.47219 | 1849.05283 |
| 316 | 1266.89974 | 2343.21663 | 26729.25 | 27734.23 | 1708.3432 | 1872.07145 |
| 326 | 994.66501 | 2085.53201 | 26652.08 | 27657 | 1627.42014 | 1851.91876 |
| 336 | 939.93256 | 1792.26982 | 26634.17 | 27635.69 | 1591.25929 | 1805.00477 |
| 346 | 944.84809 | 1584.198 | 26627.23 | 27625.6 | 1606.16988 | 1762.89161 |
| 356 | 1144.13833 | 1230.64505 | 26720.87 | 27713.9 | 1639.16517 | 1682.4972 |
| 366 | 984.19346 | 1307.05271 | 26652.88 | 27653.53 | 1612.30101 | 1703.80347 |
| 376 | 774.70724 | 1438.43364 | 26575.97 | 27573.31 | 1562.85694 | 1762.0254 |
| 386 | 490.15801 | 1677.90695 | 26477.61 | 27452.98 | 1463.69919 | 1883.98673 |
| 396 | 1107.32006 | 1024.33704 | 26777.61 | 27726.72 | 1741.32736 | 1730.18258 |
| 406 | 576.92394 | 1543.76057 | 26517.63 | 27496.69 | 1539.77529 | 1858.57408 |
| 416 | 473.9965 | 1624.80564 | 26471.89 | 27467.89 | 1449.23755 | 1884 |
| 426 | 490.78215 | 1556.0916 | 26501 | 27493.46 | 1464.17583 | 1884 |

**Table S2** Temperature working range of the optical thermometer, together with the fitting parameters obtained from the calibration curves: intercept (a) and slope (b, absolute sensitivity) for the linear model (y = a + bx), and quadratic term (c) for the case of the second order polynomial (y = a + bx + cx^2^). The coefficient of determination (R^2^) is also reported.

| Working range (K) | Thermometric Parameter | Variable | Value |
| --- | --- | --- | --- |
| 90-240 | *A_eff_* | *a* [a.u] | 1015.81 ± 25.40 |
|  |  | *b* [a.u./K] | 5.13 ± 0.13 |
|  |  | *r^2^* | 0.995 |
| 270-366 |  | *a*[a.u] | 18740.9± 1269.3 |
|  |  | *b*[a.u./K] | -95.10±8.06 |
|  |  | *c*[a.u./K^2^] | 0.126±0.012 |
|  |  | *R^2^* | 0.996 |
| 100-270 | *E_eff_* | *a* [a.u] | 26918.23 ± 13.85 |
|  |  | *b* [a.u./K] | 2.36 ± 0.06 |
|  |  | *r^2^* | 0.9 |
| 270-366 |  | *a* [a.u] | 28490.93 ± 50.19 |
|  |  | *b* [a.u./K] | -3.52 ± 0.16 |
|  |  | *r^2^* | 0.991 |

**Table S3**. MLR related fit parameters. Temperature working range, slopes (β_i_) associated to A_eff_ and E_eff_ parameters, intercept (β_0_) and the square of the coefficient of multiple correlation (R^2^). The weighted value of β_i_ is the relative weight of the slopes (unitless).

| Working range (K) | Fit parameter | *β value* | *β weight* | *R^2^* |
| --- | --- | --- | --- | --- |
| 100-240 | $\boldsymbol{\beta}_{\boldsymbol{0}}$ (K) | -7609 ± 1436 | - | 0.994 |
|  | $\boldsymbol{\beta}_{\boldsymbol{A}_{\boldsymbol{eff}}}$ (K/a.u.) | 0.054±0.027 | 0.276 |  |
|  | $\boldsymbol{\beta}_{\boldsymbol{E}_{\boldsymbol{eff}}}$ (K/cm^-1^) | 0.281±0.054 | 0.724 |  |
| 270-346 | $\boldsymbol{\beta}_{\boldsymbol{0}}$ (K) | 3717.44 ± 810.57 | - | 0.996 |
|  | $\boldsymbol{\beta}_{\boldsymbol{A}_{\boldsymbol{eff}}}$ (K/a.u.) | -0.030±0.006 | 0.540 |  |
|  | $\boldsymbol{\beta}_{\boldsymbol{E}_{\boldsymbol{eff}}}$ (K/cm^-1^) | -0.12±0.03 | 0.460 |  |

**Table S4** Fitting parameters for the temperature-dependent thermometric model.

| Parameter | Fitting function | *R*^2^ |
| --- | --- | --- |
| *τ*_ave_ | y = −1/47260.8 + 15.9×exp(31.4/(0.69*T*) − 666.6×exp(−870.3/(0.69*T*)) | 0.999 |
| *τ*_1_ | y = 1/4.3 + 1.7×exp(78.9/(0.69*T*)) − 407.4×exp(−1511.1/(0.69*T*)) | 0.999 |
| *τ*_2_ | y = −1/140.6 + 5505.6×exp(51.5/(0.69*T*)) − 6009.9×exp(−71.5/(0.69*T*)) | 0.999 |
| *A*_1_/*A*_2_ | y = −9.6 + 42.5/(1 + (*T*/208.3)^1.8^) | 0.993 |

**Figure S1** Particle size distribution of SrB_4_O_7_:Yb^2+^. Over 100 particles were measured using ImageJ, and the histogram is fitted with a Gaussian curve.

**
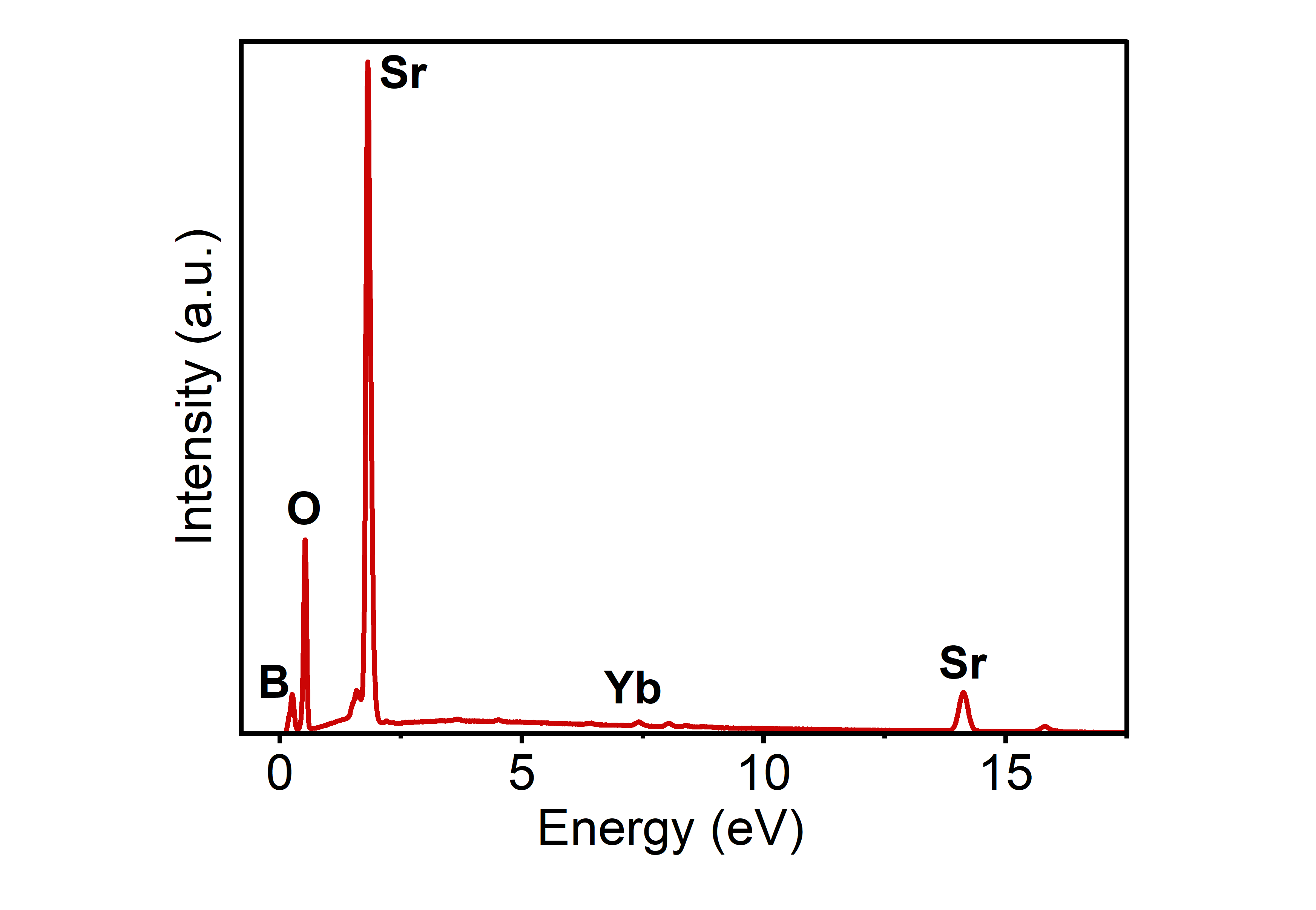
**

**Figure S2** EDX spectrum of SrB_4_O_7_:Yb^2+^

**XPS studies**

Additionally, X-ray photoelectron spectroscopy (XPS) studies were performed to further confirm the presence and stability of Yb ions in the sample (Figure S3). The high-resolution Yb 4d spectrum shows a peak at approximately 184 eV, which can be assigned to Yb^2+^, and a peak around 190 eV, corresponding to Yb^3+^,^[1,2]^ indicating that both valence states coexist in the material studied, which is in a good agreement with the absorption spectrum. A peak at ~193 eV is attributed to the B 1s signal overlapping with the Yb 4d region and is clearly labeled in the enlarged spectrum to avoid misinterpretation. The survey spectrum exhibits characteristic peaks of C 1s (~284.8 eV), O 1s (~531 eV), and Sr 3d (~134 eV). The C 1s peak is used for binding energy calibration, while the O 1s and Sr 3d peaks appear at their standard positions, confirming the reliability of the measurements.

**Figure S3** XPS spectrum of SrB_4_O_7_:Yb^2+^; the inset shows the high-resolution spectral region (200-180 eV), revealing the presence of Yb ions.

**
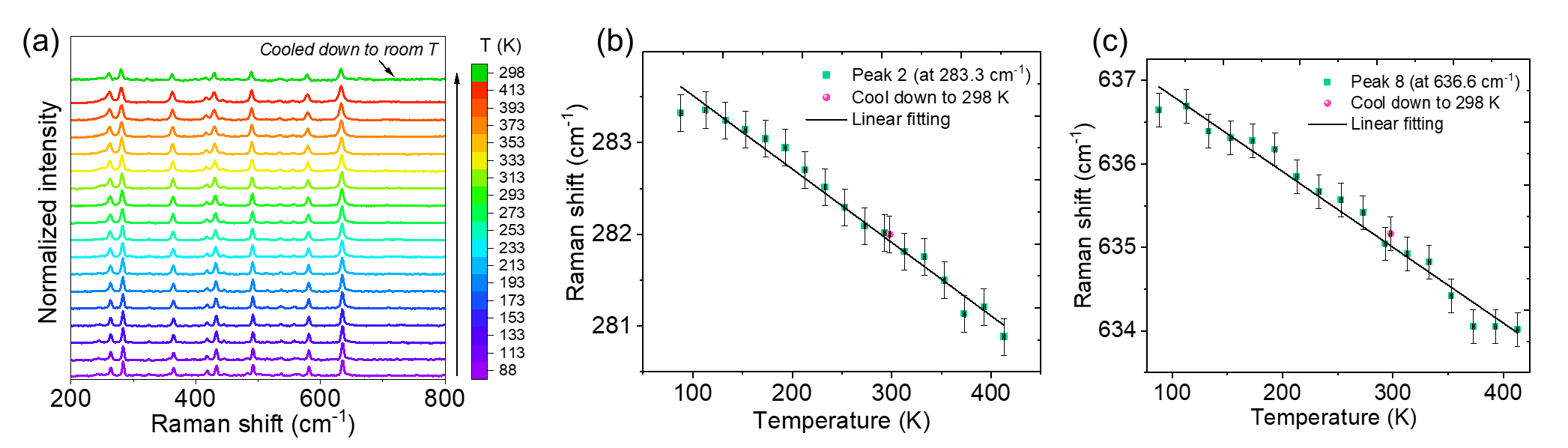
**

**Figure S4** (a) Raman spectra of the SrB_4_O_7_:Yb^2+^ measured at different temperatures during the heating process (≈80-420 K), and the last (top) spectrum collected during cooling to room temperature (298 K). (b, c) Temperature dependences of two selected, most intense Raman peaks, initially located at around 283.3 cm^-1^ (b) at 636.6 cm^-1^ (c), with the corresponding linear fits (continous lines).

**References:**

1. D.-Y. Lu, X. Wei, Q. Cai, *J. Alloys Compd*. **2021**, *884*, 161049.

2. Y. Ohno, *J. Electron Spectrosc. Relat. Phenom*. **2008**, *165*, 1.
